# Supplementary material for: An Intensive Longitudinal Assessment Approach to Surveilling Trajectories of Burnout over the First Year of the COVID Pandemic
Source: Int J Environ Res Public Health. 2023 Feb 8;20(4):2930. doi: 10.3390/ijerph20042930 (PMC9956892; doi:10.3390/ijerph20042930)
Supplement: Supplementary file 1 [file ijerph-20-02930-s001.zip › ijerph-2139351-supplementary.pdf]

International Journal of
Environmental Research and Public Health

MDPI

Supplemental Information

Analyses concerning resource needs were not included in this paper but were reported to hospital administration to incorporate emergency preparedness responses throughout the pandemic. Responses to these needed resources were scored as follows. If participants regarded the resource as a high-priority need, the response was scored as "1 = high priority", and if the participant indicated that the needed resource was a low-priority need or did not indicate that the resource was a priority at all, the resource was scored as "0—low or no priority". Initially, the three highest-priority resources identified by at least 60% of the initial 383 participants as high-priority needed resources included personal protective equipment (PPE: 78.3%), timely information (72.6%), and support and affirmation (60.3%). In the last three months of the study (from January through March 2021), 98 participants completed these questions. PPE (27.6%), timely information (21.4%), and support (20.4%) remained the resources that were most likely to be identified as high-priority needs, but the proportion of individuals regarding these as high-priority needs was much smaller. Mental health resources were seen as a high priority by 36.0% of the sample at the beginning of the study, but only 10.2% of the sample during the final three months, despite high levels of burnout.
